# Deep learning unlocks global prediction of earthquake-triggered landslides

**DOI:** 10.1093/nsr/nwaf282

**Published:** 2025-07-11

**Authors:** Filippo Catani

**Affiliations:** Machine Intelligence and Slope Stability Lab, Department of Geosciences, University of Padova, Italy

The forecasting of consequences following a strong earthquake has long been a top priority in natural risk-related modelling. However, we can reasonably state that the prediction of damage caused by landslides triggered by seismic shaking has been relatively neglected compared to other types of consequences. The reason lies in the difficulty of applying established knowledge about the local effects of earthquakes on slope stability (e.g. Newmark analysis) to the large areas typically affected by major seismic events (e.g. M > 6.0).

The most widely used forecasting tool for landslides at broad spatial scales, the susceptibility map, is generally derived from the application of statistical models that primarily use stationary data related to the physical factors predisposing slopes to instability. Seismic effects, when not entirely overlooked in susceptibility assessments, are introduced among the independent variables through parametrization linked to occurrence probability (e.g. return time) for earthquakes of a predefined magnitude. For example, in the case of subaerial landslides, one might consider the expected peak ground acceleration (PGA) for earthquakes with M > 6.5 in an attempt to incorporate into the statistical model the expected seismic shaking associated with a hypothetical scenario. A similar reasoning can also be applied to parameters related to rainfall regimes and potentially triggering precipitation.

The main limitation of these approaches from a forecasting perspective is that they provide estimates of relative susceptibility over the medium to long term, which are mostly useful for land-use planning, risk zoning, the design of major infrastructure, and the allocation of resources for risk mitigation. They are not suitable for real-time use or emergency management, as they are unable to incorporate data related to a specific triggering event.

The recent work by Fan *et al.* [1] presents, for the first time, a susceptibility assessment model that directly incorporates the real-time effects of a specific earthquake into hazard prediction. In addition to the stationary variables representing predisposing factors, it integrates the actual triggering component of an earthquake—provided that spatially explicit estimates of seismic shaking are available.

The major challenge in developing such systems is that the surface seismic effects influencing landslides are dynamic and therefore cannot be simply embedded in a frequentist statistical model as if they were static—unless a generalized seismic response estimator can be built. Fan *et al.* [1] succeed in introducing such an estimator—previously unavailable—through the careful and comprehensive construction of a dataset of nearly 400 000 landslides triggered by major earthquakes (EQTL) in the last 50 years across the globe. This dataset, which spans a wide range of geological and climatic conditions, enables the training of deep learning models with unprecedented generalization capability. As a result, Fan *et al.* [1] obtain a predictor that can swiftly produce reliable estimates of slope failure-prone areas accounting for the specific parameters of a new earthquake as soon as it occurs, once PGA data become available.

At the core of the EQTL dataset developed by Fan *et al.* [1] lies an innovative data processing workflow aimed at ensuring homogeneity in representation, mapping methods, and scale. The dataset is based on the combination of automated change detection-based mapping and careful manual correction of landslides, which was used to train a multi-scale, state-of-the-art object segmentation deep learning network. This approach builds upon the image detection methods proposed by Wang *et al.* [2] and Fang *et al.* [3] but is developed with predictive purposes rather than with the aim of mapping. This dataset represents a significant advancement over previously available datasets—such as the one compiled by Tanyaş *et al.* [4]—which, although encompassing more events, is essentially a heterogeneous aggregation of point and polygon inventories collected by different institutions, at varying scales, using diverse methods and for disparate purposes. Despite the known limitation of automated detection methods, this dataset is curated to reduce those errors to a minimum, with the purpose to build a predictive model, not to increase accuracy in mapping.

Beyond the dataset itself, the model developed by Fan *et al.* [1] is notable for its statistical framework for susceptibility estimation, which introduces several innovations compared to previous work in the literature. Among them, the deep learning (DL) framework is worthy of note, as it introduces multi-scaling feature selection and representation with a leave-one-out cross-validation strategy using 38 available seismic events. The DL model captures spatial and hierarchical patterns directly from the input data with convolutional layers acting as a set of spatial filters, automatically detecting increasingly complex features, from basic edges and shapes to sophisticated geomorphological and geological patterns that characterize landslide susceptibility, across a moving window of 448 pixels by side, at 30-m resolution. Although less inherently explainable with respect to machine learning models such as random forests (RFs), this method goes beyond RF limitations as it does not require pre-extracted features but generates new activations across the analysis window according to local landscape properties. This greatly increases the generalization capability of the model, which the authors claim to be of global applicability, and the computational time during emergencies. Being pre-trained over the extensive 38 global events for a total of ∼400 000 landslides, the model by Fan *et al.* [1] has low computational costs at forward prediction time, as the authors have tested on the unseen recent Luding 2022 earthquake's curated EQTL dataset.

Since the model by Fan *et al.* [1] already demonstrates proven applicability for predicting the effects of an earthquake on slope stability, this bodes well for further possible improvements, which could be implemented relatively easily within it, given the flexibility of the deep learning framework used.

For example, the authors apply spatial statistics of landslide factors at the 30 m scale using a fixed-size sliding window of 448 × 448 pixels. Although the dimensions of this window are reasonably sufficient to encompass all the physical characteristics that may influence the stability of the central pixel, we believe that an optimization approach using variable window sizes could further improve the granularity of the estimates, better highlighting local effects and spatial heterogeneity in slope properties. A geostatistical approach based on the average local size of landslides relative to slope units could also be helpful (see, for example, the method used by Tucker *et al.* [5]).

Another improvement is to account for the spectral characteristics of the seismic wave field. Dynamic-scale parametrization of landslide dimension relative to wavelength, such as wave-landform interactions, should be considered. This would be particularly important for large landslides exceeding in size wavelength or effective seismic energy concentration zones. Seismic energy can, under specific local conditions, average out or be less focused over large unstable slope sectors, reducing the net triggering force. Although the convolutional neural network (CNN) could learn some implicit spatial context through convolutional filters (especially in larger receptive fields), this would be limited to recognizing patterns in the co-location of high slopes and high shaking—not physics-based interactions between wave propagation and slope geometry. Some possible solutions could be to include spectral ratios or dominant frequencies at the ground when available, incorporating landslide size as a predictor, using physics-informed machine learning combining digital elevation models (DEMs) with simulated seismic wavefields, and using dynamic rupture modeling to capture local slope responses.

Further improvements, beyond the current capabilities of the model, could involve (i) the inclusion of landslide volume as a model output—a step that would require enhancements in curated landslide-volume datasets and predictive statistics, (ii) the modeling of runout dynamics, (iii) the explicit integration of long-term seismic ‘memory’ effects to enable scenario-based forecasting and temporal evolution analyses, and (iv) the creation of a parallel prediction system for rainfall-induced landslides. Such a system would ideally complement the current earthquake-triggered model and could be developed by slightly adapting the existing deep learning framework and building upon a curated collection and pre-processing of available global datasets on landslide-triggering rainfall events.
